# Understanding Deep Brain Stimulation:* In Vivo* Metabolic Consequences of the Electrode Insertional Effect

**DOI:** 10.1155/2018/8560232

**Published:** 2018-10-17

**Authors:** Marta Casquero-Veiga, David García-García, Manuel Desco, María Luisa Soto-Montenegro

**Affiliations:** ^1^Instituto de Investigación Sanitaria Gregorio Marañón, Madrid 28007, Spain; ^2^CIBER de Salud Mental (CIBERSAM), Madrid 28029, Spain; ^3^Departamento de Bioingeniería e Ingeniería Aeroespacial, Universidad Carlos III de Madrid, Leganés 28911, Spain; ^4^Centro Nacional de Investigaciones Cardiovasculares (CNIC), Madrid 28029, Spain

## Abstract

Deep brain stimulation (DBS) is a neurosurgery technique widely used in movement disorders, although its mechanism of action remains unclear. In fact, apart from the stimulation itself, the mechanical insertion of the electrode may play a crucial role. Here we aimed to distinguish between the insertional and the DBS effects on brain glucose metabolism. To this end, electrodes were implanted targeting the medial prefrontal cortex in five adult male Wistar rats. Positron Emission Tomography (PET) studies were performed before surgery (D0) and seven (D7) and nine days (D9) after that. DBS was applied during the ^18^FDG uptake of the D9 study. PET data were analysed with statistical parametric mapping. We found an electrode insertional effect in cortical areas, while DBS resulted in a more widespread metabolic pattern. The consequences of simultaneous electrode and DBS factors revealed a combination of both effects. Therefore, the insertion metabolic effects differed from the stimulation ones, which should be considered when assessing DBS protocols.

## 1. Introduction

In recent years, brain stimulation techniques have emerged in bioscientific and clinical scenarios. Deep brain stimulation (DBS) is a technique that modulates neuronal discharge patterns through electrical current both locally, at the electrode implantation site, and also in remote brain areas associated with the deep brain target [1, 2]. The success and safety offered by DBS in movement disorders [3] have led to consider its potential application in other neurological and mental pathologies, such as psychiatric disorders [4–6], with the subsequent search for new DBS targets. However, the mechanism of action of DBS remains unclear and depends on two confounded factors: the electrode insertion* per se* and the electrical stimulation. Indeed, certain symptomatology improvement has been related to the mere insertion of the electrodes in the treatment of epilepsy [7] and chronic neuropathic pain in humans [8]. Also, antidepressant-like effects have been found in rats in which electrodes were implanted, but without applying electrical stimulation [9]. To our knowledge, these are the only studies that have shown this insertional effect, but none of them has studied the subsequent brain regional activity modulation. Thus, the aim of this study is to assess the insertional effect of the electrode, isolated from the acute electrical stimulation itself, on brain glucose metabolism studied by positron emission tomography (PET) and statistical parametric mapping (SPM) techniques in rats with electrodes placed in the medial prefrontal cortex (mPFC).

## 2. Materials and Methods

### 2.1. Animals

Adult male Wistar rats (~ 350 g) (N = 5) were housed in a temperature- and humidity-controlled vivarium, on a 12h light-dark cycle, with standard laboratory rat chow and water ad libitum. Animals were deprived of food 6-8 hours prior to the PET study. All experimental animal procedures were conducted according to the European Communities Council Directive 2010/63/EU and approved by the Ethics Committee for Animal Experimentation of the Hospital Gregorio Marañón.

This study was performed following the guidelines established by the principles of the 3Rs to minimize the number of animals included in this work [11]. Nevertheless, considering the longitudinal design of this research, the number of animals was sufficient to obtain enough statistically significant differences between time points.

### 2.2. Stereotaxic Surgery and DBS Protocol

Animals were anesthetized with ketamine/xylazine (100/10 mg/kg). Concentric bipolar platinum-iridium electrodes (Plastics One, Roanoke, USA) were bilaterally implanted targeting the mPFC (+3.5mm posterior, +0.6mm lateral from Bregma, -3.4mm ventral from Dura) [10]. Electrodes were fixed to the skull surface with dental acrylic cement (Technovit®, Germany). Antibiotic (ceftriaxone, 100mg/kg) and analgesic (buprenorphine, 0.1mg/kg) drugs were administered for 3 days as postoperative care.

DBS was applied during the radiotracer uptake period (45 min) with an isolated stimulator (CS 120 8i, CIBERTEC S.A., Spain) in a constant current mode at 130 Hz, 150 *μ*A and a pulse width of 100 *μ*s.

### 2.3. Imaging Studies

**Figure 1:**
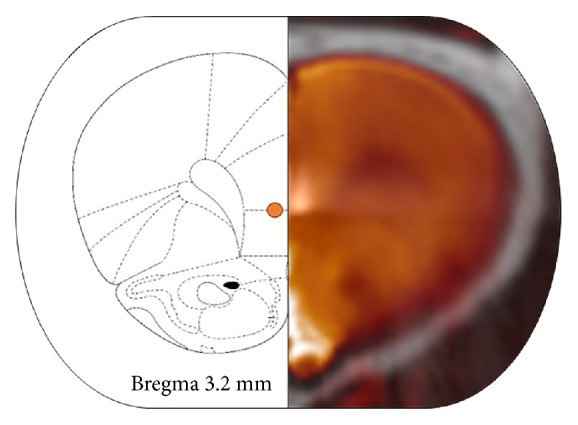
Electrode placement. Axial view of a CT scan registered to the MR template of an animal (right) and its correspondent slice in the Paxinos & Watson atlas [10] (left), to verify the correct electrode location in the mPFC. The bright and orange points represent the electrode tip in the CT and atlas images, respectively.

PET and computerized tomography (CT) scans were acquired just before surgery (D0, baseline) and 7 days (D7, without stimulation) and 9 days (D9, with stimulation) after that, in order to provide enough time for surgical recovery [12]. Additional CT scans were acquired at the end of the surgery to verify the correct placement of the electrodes (Figure 1). In addition, one magnetic resonance imaging (MRI) scan of a single nonoperated animal was acquired to be used as an anatomical template.

Animals were scanned using a small-animal PET/CT scanner (ARGUS PET/CT, SEDECAL, Madrid), under anaesthesia with isoflurane (3% induction, 1.5% maintenance in 100% O2). 2-Deoxy-2-[^18^F]fluoro-D-glucose (FDG,** ~ **37Mbq) was intravenously injected and, after 45 min of uptake, animals were scanned for 40 min. Images were reconstructed using a 2D-OSEM algorithm, with a spatial resolution of 1.45 mm Full Width Half Maximum (FWHM), a voxel size of 0.3875 x 0.3875 x 0.775 mm^3^, and an energy window of 400-700 keV. Decay and dead-time corrections were applied.

CT studies were acquired with the same scanner, using the following parameters: 340 mA, 40 kV, 360 projections, 8 shots, and pixel size of 200 *μ*m. Images were reconstructed using an FDK algorithm (isotropic voxel size of 0.124 mm) [13].

The MRI study was acquired with a 7-Tesla Biospec 70/20 scanner (Bruker, Ettlingen, Germany). A T2-weighted spin-echo sequence was acquired with TE=33 ms, TR=3732 ms, and a slice thickness of 0.8 mm (34 slices). The matrix size was 256 × 256 pixels with a FOV of 3.5 × 3.5 cm2.

### 2.4. Analysis of PET Data

PET images postprocessing and voxel value normalization were performed following the protocols previously described by our group [14, 15]. Briefly, PET images were spatially coregistered to a random reference CT scan (CTref) and smoothed with an isotropic Gaussian kernel of 2 mm FWHM. A brain mask segmented in the MRI, also registered to the CTref was applied to all PETs to exclude voxels outside the brain. Voxel values were normalized to average intensity of a brain region without statistically significant differences between groups [15].

The statistical analysis consisted on a voxel-wise analysis of PET data using SPM12 (http://www.fil.ion.ucl.ac.uk/spm/software/spm12/) by means of paired T-tests, setting a significance threshold of p<0.005 uncorrected (voxel-level significance), but cluster-based corrected in order to avoid type II errors [16]. Only clusters higher than 50 adjacent voxels were considered aiming at reducing type I error.

We performed three different comparisons to evaluate the modulatory effect of the electrode insertion (D0 versus D7, study I), the stimulation (D7 versus D9, study II), and the combination of both the insertion and the stimulation (D0 versus D9, study III), on brain metabolism.

In this sense, we assume that the metabolic differences we show in the study II are almost completely due to the acute effect of the high-frequency electrical stimulation. Although the microlesional effect related to the electrode insertion is highly variable between subjects [8], the stimulation effect has been shown to be much stronger that the insertional one [17], and this latter tends to reduce over time. Furthermore, both the insertion (D7) and the stimulation (D9) PET acquisitions were separated by just two days, period in which no new relevant consequences derived from the electrode presence are expected. In this context, although a late effect of the insertion could have appeared, its influence on the study II would be minimum and possibly masked by the impact of a stronger stimulus represented by the application of high-frequency electrical stimulation.

## 3. Results 

### 3.1. Electrode Insertion Effect

**Figure 2:**
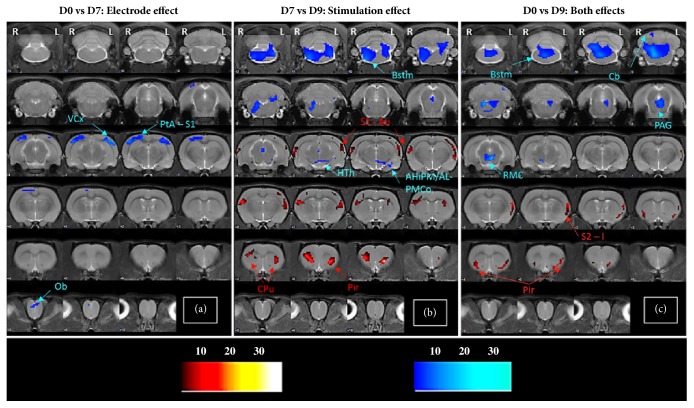
Changes in brain metabolic activity. Voxel based SPM results in T-maps overlaid on a T2 MR image, showing the changes in glucose metabolism due to electrodes insertion (a), stimulation (b), and both effects (c). The color bars in the right represent the T values corresponding to lower (blue) and higher (red) FDG uptake (p-value <0.005 (unc.); k= 50 voxels). Glucose metabolism: increase (hot colors); decrease (cold colors) [AHiPM/AL: amydalohippocampal area posteromedial/anterolateral part, Au: auditory cortex, Bstm: brainstem, Cb: cerebellum, CPu: caudate-putamen, HTh: hypothalamus, I: insular cortex, Ob: olfactory bulb, PAG: periaqueductal grey matter, Pir: piriform cortex, PMCo: posteromedial cortical amygdaloid nucleus, PtA: parietal association cortex, RMC: red nucleus, S1: primary somatosensory cortex, S2: secondary somatosensory cortex, and VCx: visual cortex].

The presence of the electrodes (D0 versus D7, study I) led to a reduced FDG uptake in parietal association (PtA), primary somatosensory (S1), and visual cortices (Figure 2(a); Table 1(A)).

### 3.2. Stimulation Effect

The electrical stimulation (D7 versus D9, study II) led to a decreased FDG uptake in brainstem (Bstm), amygdaloid nuclei (AHiAL, AHiPM, and PMCo), and hypothalamus (HTh), together with an increased metabolism in caudate-putamen (CPu), piriform (Pir), S1, and auditory cortex (Au) (Figure 2(b); Table 1(B)).

### 3.3. Insertion and Stimulation Effect

The combination of both effects (D0 versus D9, study III) showed a decreased FDG uptake in Bstm, red nucleus (RMC), and periaqueductal grey matter (PAG) and higher metabolism in secondary somatosensory (S2), insular (I), and Pir cortices (Figure 2(c), Table 1(C)).

## 4. Discussion

First, we describe an insertional effect on brain glucose metabolism in sensory areas that are connected to mPFC [18]. Second, mPFC-DBS resulted in a distinct brain metabolic pattern, with more brain areas affected than in study I. Thus, DBS induced changes in circuits where the mPFC plays a key role, such as limbic (AHi, PMCo, and Pir) and reward (CPu and Bstm) systems [18]. Finally, the simultaneous consequences of the electrodes and the stimulation revealed lower cortical activation compared to the study II, showing a compensation of the hypometabolism derived from the electrodes presence (study I). Specifically, the absence of metabolic changes in S1 shown in study III exemplifies this mechanism, as this structure showed a metabolic reduction and an increase in studies I and II, respectively. Moreover, S1 is the only region in which there is an overlap between both effects.

The insertion effect could appear in response to the microlesion induced by the electrode in the mPFC [7] and the subsequent inflammation of the targeted area [9]. Thus, although the microlesion effect fades away over time [19], the clinical manifestations of the insertional effect could persist from days to months (exceptionally, years), or even being absent, despite comparing patients under the same surgical protocol and disease [8]. Besides, other authors have also provided evidence of its permanence on the healthy rat brain metabolism beyond one week after surgery [12]; although, in contrast to our findings, they showed similar effects of stimulation and insertion, being the latter of lower intensity. Furthermore, comparable results have been also shown in Parkinson disease (PD) patients after electrode insertion in the subthalamic nucleus, which resulted in similar but lower metabolic changes than subthalamotomy in PD-related pattern, while no significant clinical effect was observed due to the insertion [20]. Conversely, task-fMRI data found partial differences between the insertion and stimulation consequences in PD [19].

Therefore, the wide variability showed in relation to the clinical and physiological consequences of the electrode insertion could be highly dependent on several factors (e.g., the health state of the subjects, the DBS target selected, the number of microelectrode recording trajectories performed during the surgery [20], the time elapsed between the surgery and the test, etc.). In fact, PFC input and output connections shared with the sensory cortex occupy different locations and ordering [21], which is not common to other regions and could suppose a substantial difference regarding DBS effect. Thus, the opposed metabolism caused in the somatosensory cortex by the electrode placement and the stimulation alone could respond to the recent neural informative disruption theories of DBS mechanism of action [22, 23]. Importantly, these changes would have not been uncovered without a 3-times longitudinal design. Taken all together, both stimulation and insertion results seem to involve the same brain networks, although in a considerably different extent. This information would be helpful for adjusting the DBS protocols. Thus, understanding the regions affected by each involved factor (insertion; stimulation), together with the intensity and direction (activation; inhibition) of the produced modulation, could lead to more specific and efficient DBS protocols.

## 5. Limitations

Our work is subjected to several limitations. On the one hand, the small sample size selected, which responds to the aim of providing preliminary evidences that we considered to be important for understanding the DBS mechanism of action through a metabolic perspective. Therefore, we applied strict statistical thresholds in order to show more accurate results, which lead us to discard potentially important effects that do not reach statistical significance. In fact, the electrode insertion has been proved to produce lower metabolic changes than subthalamotomy or stimulation [12, 20], which could be masked in the present study. These points would explain why, in contrast with previous literature, the metabolic pattern observed with the insertion differs from that caused by the stimulation.

On the other hand, we have only included healthy animals in our study, which does not allow us to extrapolate the observed changes to a disease model due to the differences related to a diseased brain. Nevertheless, we aimed to describe the metabolic consequences of electrode insertion and electrical stimulation, excluding any other intervening factors, in order to isolate those effects and improve their metabolic characterization.

## 6. Conclusions

In conclusion, our study highlights the importance of the design of appropriate protocols, particularly in neuroimaging, emphasizing the value of scanning the same subject with/without DBS, for a full understanding of the DBS mechanism of action and its clinical consequences. This will allow taking advantage of the electrodes and the stimulation consequences in order to optimize the DBS protocols for achieving the desired therapeutic effects.

## Figures and Tables

**Table 1:** Changes in brain metabolism due to electrode (A), stimulation (B), and both effects (C).

**ROI**	**Side**	**T**	**k**	**↓/↑**	**p**_**unc**_ ** peak level**	**FWE** **peak level**	**FWE** **cluster level**
**(A) D0 vs D7: Electrode effect**							

Ob	R & L	15.68	121	**↓**	<0.001	0.811	0.067

PtA - S1	R	14.97	365	**↓**	<0.001	0.880	<0.001

VCx	L	14.75	184	**↓**	<0.001	0.884	0.015

**(B) D7 vs D9: Stimulation effect**							

Bstm	R & L	18.39	1549	**↓**	<0.001	0.432	<0.001
AHiPM/AL-PMCo - HTh	L	10.39		**↓**	<0.001	0.949	

CPu	L	37.56	738	**↑**	<0.001	0.025	<0.001
S1-Au		10.53		**↑**	<0.001	0.947	

CPu-Pir	R	17.74	695	**↑**	<0.001	0.497	<0.001
S1-Au		10.45		**↑**	<0.001	0.948	

**(C) D0 vs D9: Both effects**							

RMC - PAG	R & L	26.24	1430	**↓**	<0.001	0.105	<0.001
Cb	R	5.90		**↓**	0.002	0.998	

S2 - I	L	15.20	475	**↑**	<0.001	0.892	<0.001
Pir	L	9.10		**↑**	<0.001	0.979	

Pir	R	12.96	152	**↑**	<0.001	0.929	0.026

Structures: AHiPM/AL: amydalohippocampal area posteromedial/anterolateral part, Au: auditory cortex, Bstm: brainstem, Cb: cerebellum, CPu: caudate-putamen, HTh: hypothalamus, I: insular cortex, Ob: olfactory bulb, PAG: periaqueductal gray matter, Pir: piriform cortex, PMCo: posteromedial cortical amygdaloid nucleus, PtA: parietal association cortex, RMC: red nucleus, S1: primary somatosensory cortex, S2: secondary somatosensory cortex, and VCx: visual cortex.

ROI: region of interest. Side: right (R) and left (L). T: t value; k: cluster size. Glucose metabolism: increase (**↑**) and decrease (**↓**). p_unc_: p-value uncorrected; FWE: family wise error correction.

## Data Availability

The data used to support the findings of this study are included within the article.
